# Pott's Puffy Tumor: Intracranial Extension Not Requiring Neurosurgical Intervention

**DOI:** 10.7759/cureus.10106

**Published:** 2020-08-29

**Authors:** Michael W Song, Margaret Montovano, Aleksander Kubiak, Shaza Khalid, Jerrold Ellner

**Affiliations:** 1 Infectious Diseases, Rutgers New Jersey Medical School, Newark, USA; 2 Radiology, Rutgers New Jersey Medical School, Newark, USA

**Keywords:** pott's puffy tumor, intracranial epidural abscess, functional endoscopic sinus surgery, leptomeningeal enhancement, acute sinusitis

## Abstract

Pott’s puffy tumor, typified by a subperiosteal abscess underlying the frontal bone, is an uncommonly encountered clinical entity that can occur in the setting of local trauma or secondary to frontal sinusitis. Diagnosis can be challenging, as cultures may be sterile, and the condition must be differentiated from neoplasm and superficial and soft tissue infection. Although more common in the pediatric population, Pott’s puffy tumor must remain on the differential with a high index of suspicion in adult patients who fit the clinical picture. Early diagnosis with CT or MRI and therapeutic medical and surgical intervention are crucial as intracranial complications, such as abscess and empyema can occur and may be fatal. We present an adult patient with a history of inhaled drug abuse who presented with Pott’s puffy tumor with meningitis and bifrontal epidural abscesses at presentation. There is evidence in the literature that management of sinus-related intracranial epidural abscess with antibiotic therapy and adequate surgical or endoscopic surgical drainage may bypass the need for neurosurgical intervention, as was the case here.

## Introduction

Pott’s puffy tumor is osteomyelitis of the frontal bone accompanied by abscess in the subperiosteal region. Generally occurring secondary to sinusitis or trauma, this rare clinical entity and its intracranial complications develop due to the anatomy of the sinuses and their venous drainage [1,2]. The diploic veins responsible for draining the frontal sinus communicate directly with the dural venous sinuses, thereby allowing hematogenous spread of the infection. Purulent erosion through the frontal bone results in the development of the described subperiosteal abscess; further inward extension can result in intracranial complications, such as epidural or subdural empyema, meningitis, or abscess [2].

We present a case of Pott’s puffy tumor in an adult patient complicated by pachymeningitis and leptomeningitis and bifrontal epidural abscesses. 

## Case presentation

A 31-year-old man with a history of inhaled heroin and fentanyl abuse presented to our hospital with a one-week history of progressive headache and pain over his left eye with associated swelling and subjective fevers. The patient also reported several days of weakness, fatigue, and poor oral intake but denied any vision changes or eye discharge. He denied any history of recent trauma and denied any other medical or surgical history.

**Figure 1:**
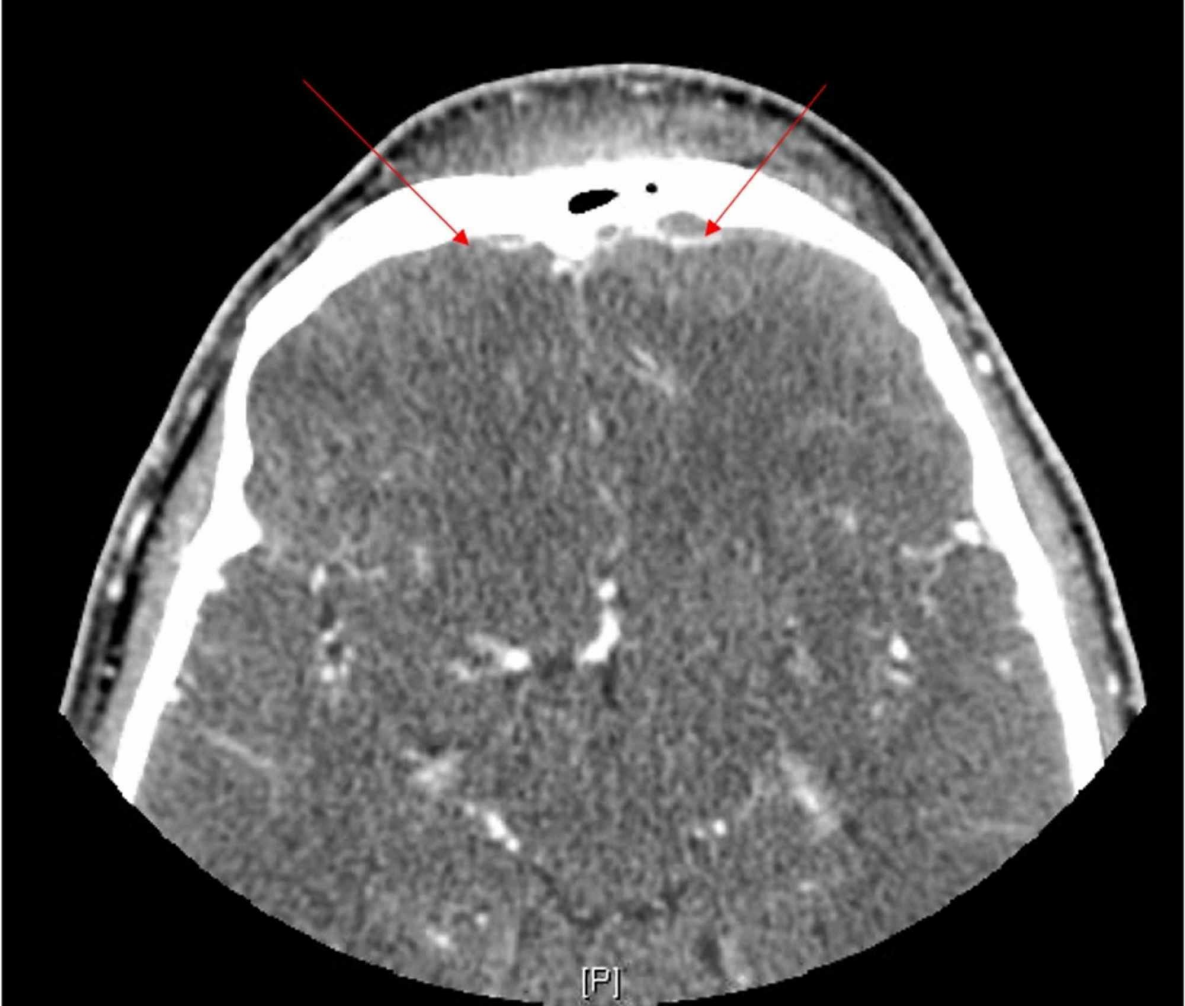
CT head with contrast shows swelling and enhancement of the front scalp. There are peripherally enhancing extra-axial abscesses subjacent to the frontal bones (arrows).

In the ED, the patient was febrile to 102.5˚F. Physical exam revealed an erythematous left eye with significant periorbital swelling; the patient was unable to open the affected eye. Labs were significant for a leukocytosis of 17.4 x 10^3^ cells/μL. CT scan of the orbits revealed preseptal and orbital cellulitis with evidence of intracranial extension of the infection with enhancing wall fluid collections suggestive of bifrontal epidural abscesses secondary to frontal sinusitis. Diffuse soft tissue swelling in the bifrontal scalp was consistent with Pott’s puffy tumor (Figure 1).

**Figure 2:**
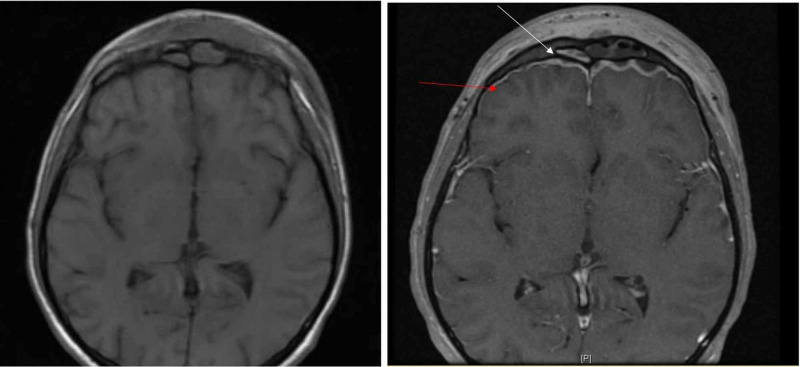
MRI T1-weighted pre- and post-contrast axials. Abscess is seen within the left frontal sinus (white arrow). Abscesses are seen underlying the bilateral frontal bones. Again seen is enhancement of the edematous frontal scalp compatible with Pott's puffy tumor. There is bilateral frontal pachymeningeal and leptomeningeal enhancement compatible with meningitis (red arrow).

Infectious diseases, ENT (ear, nose, throat), neurosurgery, and ophthalmology were consulted. Further workup with MRI of the brain with and without contrast was performed and demonstrated left periorbital and left frontal subcutaneous soft tissue cellulitis and edema consistent with Pott’s puffy tumor as well as bifrontal pachymeningitis and leptomeningitis and sinusitis with abscess formation in the frontal sinus, left maxillary sinus, anterior left ethmoid sinus, and the bifrontal epidural space (Figure 2).

Blood cultures from admission remained sterile. Per ENT, operative intervention was delayed for 72 hours of antibiotic therapy with IV vancomycin 1.25 g every eight hours, cefepime 2 g every eight hours, and metronidazole 500 mg every eight hours; the patient then underwent frontal bilateral sinusotomy, total ethmoidectomy, sphenoidotomy, maxillary antrostomy, and inferior turbinate reduction. Surgical pathology revealed sinonasal mucosa with chronic inflammation, cartilage, and reactive bone. Postoperative CT head with and without contrast revealed decreased left periorbital and frontal cellulitis and bifrontal epidural abscesses that were stable in size with no evidence of mass effect. Per neurosurgery, no acute surgical intervention was necessary for drainage of residual epidural abscesses.

Two days postoperatively, the patient signed out against medical advice and was discharged on a six-week course of oral levofloxacin 750 mg one tab daily, linezolid 600 mg one tab daily, and metronidazole 500 mg one tab daily.

Three weeks later, the patient returned for follow-up in the outpatient clinic. He complained of a mild headache controlled with medication and otherwise reported doing well. The patient was afebrile with a white count of 7.2 x 10^3^ cells/μL; physical exam revealed no forehead or periorbital edema and intact extraocular movements. Repeat CT with contrast showed decreased size of the bifrontal epidural abscesses and improved aeration of the sphenoid and maxillary sinuses.

## Discussion

Pott’s puffy tumor is an infrequently encountered clinical entity in the setting of improved antibiotic treatment for sinusitis. Although it can be associated with local trauma, Pott’s puffy tumor generally arises secondary to untreated or partially treated frontal sinusitis [1]. While it can occur in any population, it is generally seen in adolescent patients as adolescence coincides with increased vascularity and growth of the frontal sinuses [3]. Microbiological diagnosis can be challenging, as cultures may be negative in up to 50% of cases. When a pathogen is identified, the most common causes are Staphylococcus aureus, Streptococcus species, and oral anaerobes [4,5].

Typical presentation of Pott’s puffy tumor includes symptoms of fever, headache, periorbital swelling and pain, purulent rhinorrhea, and vomiting. The clinical picture must be differentiated from more common conditions, such as neoplasm and superficial and soft tissue infection. Due to the rarity of Pott's puffy tumor, which is even more pronounced in adult patients such as ours, diagnosis is often challenging and can be delayed, leading to life-threatening complications. Intracranial complications can arise from direct extension or venous drainage and include subdural, epidural, and intraparenchymal abscesses, meningitis, and cavernous sinus thrombosis [4]. While these complications may occur at a high rate of 60% or higher in pediatric and adolescent patients, adult patients appear to develop intracranial complications less frequently at a rate of approximately 29% according to prior reports [6]. Nonetheless, as is evidenced by our case, additional workup for intracranial complications (i.e. MRI) should be performed in adult patients with Pott’s puffy tumor, even when neurologically asymptomatic. Although our patient had pachymeningitis and leptomeningitis and bifrontal epidural abscesses, there was no evidence of mass effect on imaging or signs of increased intracranial pressure or focal neurological deficits on examination. Prior reports of Pott’s puffy tumor have demonstrated that intracranial complications are often asymptomatic when the abscess is located in a silent area of the central nervous system [7].

Because of its rarity and potentially fatal complications, Pott’s puffy tumor should be on the differential in patients with painful forehead swelling and frontal sinusitis or recent trauma. Diagnosis must be discerned from benign or malignant neoplasm, soft tissue infection, or hematoma with superimposed infection. CT or MRI confirms the diagnosis. Combined medical treatment with antibiotics and surgical treatment with drainage and debridement or endoscopic intervention is the standard of care [4]. As is demonstrated by our case, even when intracranial complications such as epidural abscesses exist, prompt attention to the sinuses with surgical or endoscopic drainage combined with intravenous antibiotics may decrease the need for neurosurgical intervention. A prior study by Heran et al. demonstrated that sinus-related intracranial epidural abscesses could be managed without neurosurgical intervention in the setting of minimal mass effect from the abscess and adequate endoscopic or surgical sinus drainage; eight children who fit these criteria received antibiotic therapy specific to the identified organisms and all experienced radiologic and clinical resolution of the sinus-related intracranial epidural abscess [8]. In the setting of improved techniques for sinus drainage and guided medical therapy, avoiding neurosurgical intervention may be a viable therapeutic option in the management of future cases similar to ours.

## Conclusions

A high index of suspicion is necessary to recognize Pott’s puffy tumor and its potentially fatal intracranial complications, especially in adult patients such as ours whose complications may be neurologically asymptomatic. Early diagnosis and therapeutic intervention for Pott’s puffy tumor are crucial to increase the likelihood of favorable outcomes.

Diagnosis can be established with CT with contrast followed with MRI of the brain to delineate the extent of intracranial complications, as was performed in our case. Therapeutic intervention is generally pursued in the form of combined medical and surgical management; a case such as ours demonstrates that intravenous antibiotic therapy with prompt surgical or endoscopic drainage of the sinuses may allow for conservative management of intracranial epidural abscesses from a neurosurgical perspective.
